# Spontaneous Resolution of Abdominal Pseudohernia Following Lung Cancer Surgery: A Case Report

**DOI:** 10.7759/cureus.64250

**Published:** 2024-07-10

**Authors:** Maiya Chen, Yujo Kawashita, Sosei Abe, Takashi Ueda, Junzo Yamaguchi

**Affiliations:** 1 Respiratory Medicine, Fukuoka University Hospital, Fukuoka, JPN; 2 Surgery, Fukuoka Seisyukai Hospital, Fukuoka, JPN

**Keywords:** cancer immunity, abdominal wall hernia, lung cancer, herpes zoster, abdominal pseudohernia

## Abstract

Abdominal pseudohernia is a condition characterized by the protrusion of abdominal viscera through a weakened area of the abdominal wall without a hernia sac. Various causes, including spinal disorders and trauma, can lead to this condition; however, the most common cause is reported to be herpes zoster. We present a rare case of spontaneous resolution of abdominal pseudohernia following lung cancer surgery. A 71-year-old male presented with left upper abdominal bulging and pain. A CT scan performed at the time incidentally revealed a nodular lesion in the right lower lobe, suspicious for lung cancer. Single-port thoracoscopic surgery was performed, and the final diagnosis was right lower lobe lung squamous cell carcinoma. Following the lung cancer surgery, the left upper abdominal bulging spontaneously resolved within one week. In this case, we hypothesize that the immune dysregulation caused by lung cancer increased the activity of the herpes zoster virus, leading to the development of pseudohernia. The spontaneous resolution of the pseudohernia is thought to be due to the improvement of immune dysregulation after surgery.

## Introduction

An abdominal hernia is a condition in which abdominal viscera protrude through a weakened area of the abdominal wall. When a hernia sac is absent, it is called a pseudohernia. Pseudohernia can also occur as a rare motor complication of herpes zoster. Herpes zoster is caused by the reactivation of the varicella-zoster virus. This virus initially causes infection during childhood as chickenpox and becomes dormant in the dorsal root ganglia of the nerves. Once the virus is reactivated, a vesicular eruption appears along a dermatome and can cause pain. Postherpetic neuralgia is a well-known complication of herpes zoster, but motor complications are not widely recognized [1]. In this case report, we present a rare case of spontaneous resolution of abdominal pseudohernia following lung cancer surgery.

## Case presentation

**Figure 1:**
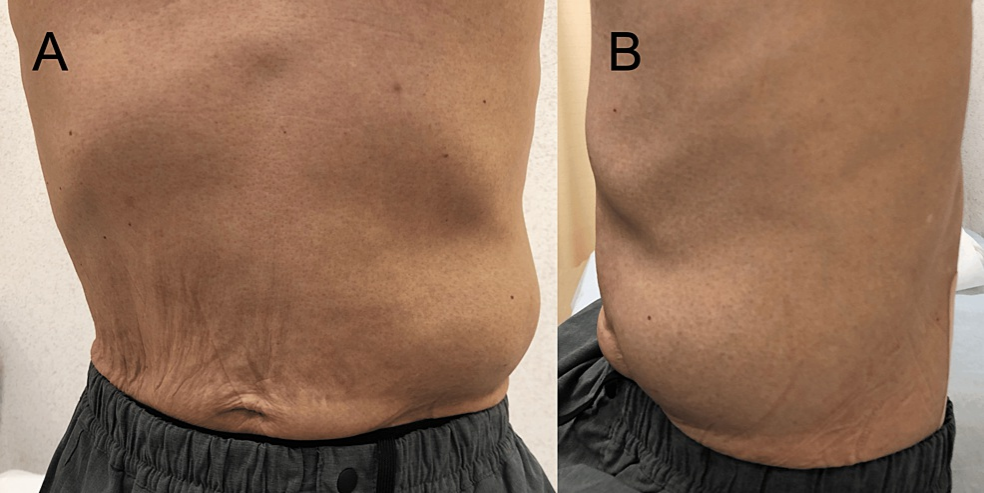
Left upper abdominal bulging. On presentation, bulging was observed in the left upper abdomen, extending to the lateral side.

**Figure 2:**
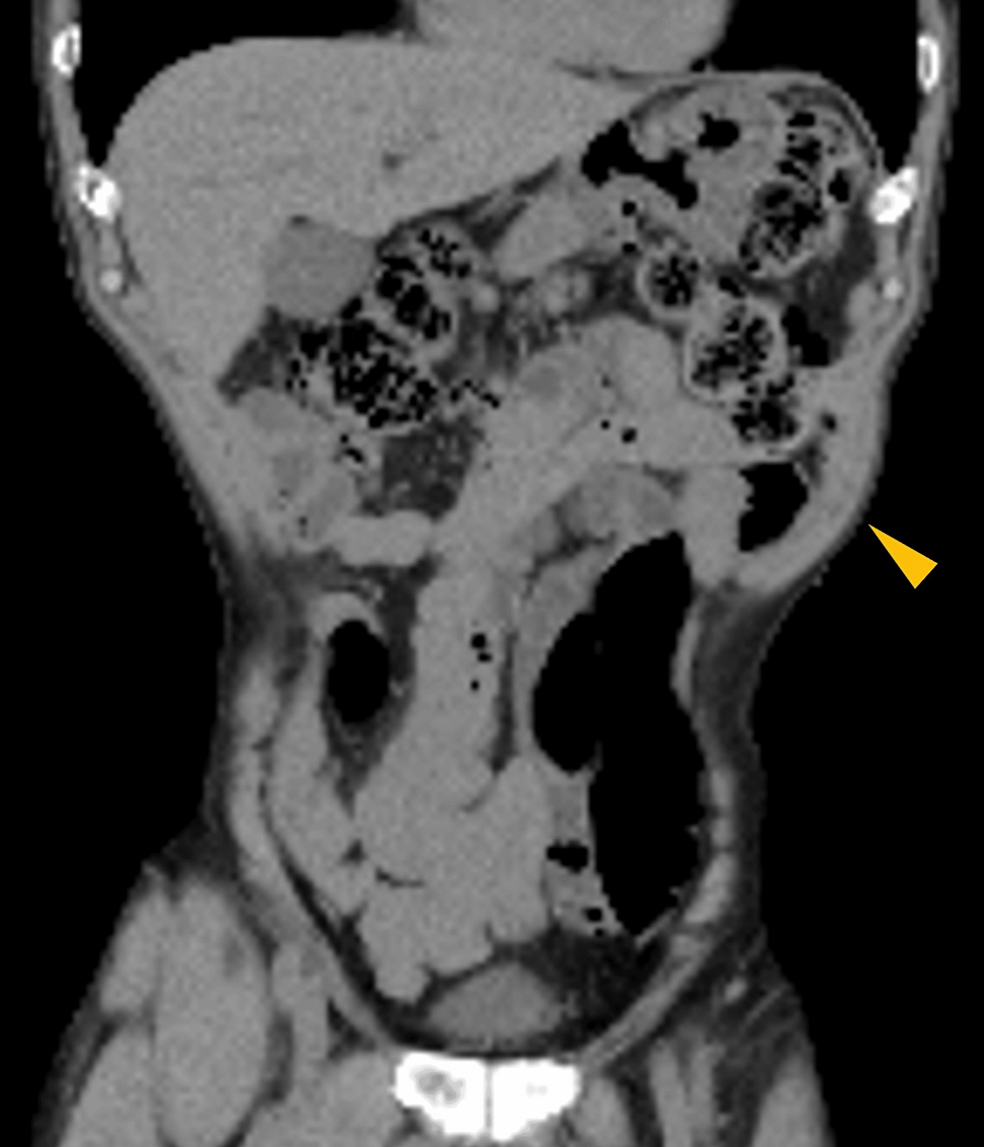
Plain abdominal CT imaging of the patient. The plain abdominal CT imaging revealed muscle relaxation in the left upper abdominal bulge area with no obvious hernia sac or hernia contents (arrow).

**Figure 3:**
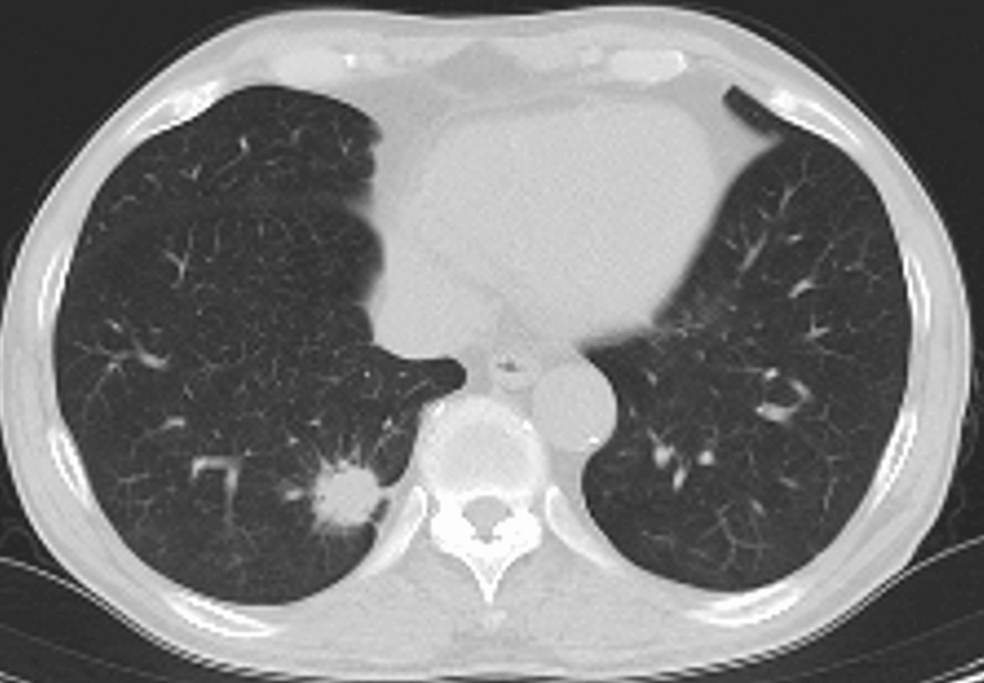
Plain chest CT imaging of the patient. Plain chest CT imaging revealed an irregular nodular shadow with spiculation in the right lower lobe.

**Figure 4:**
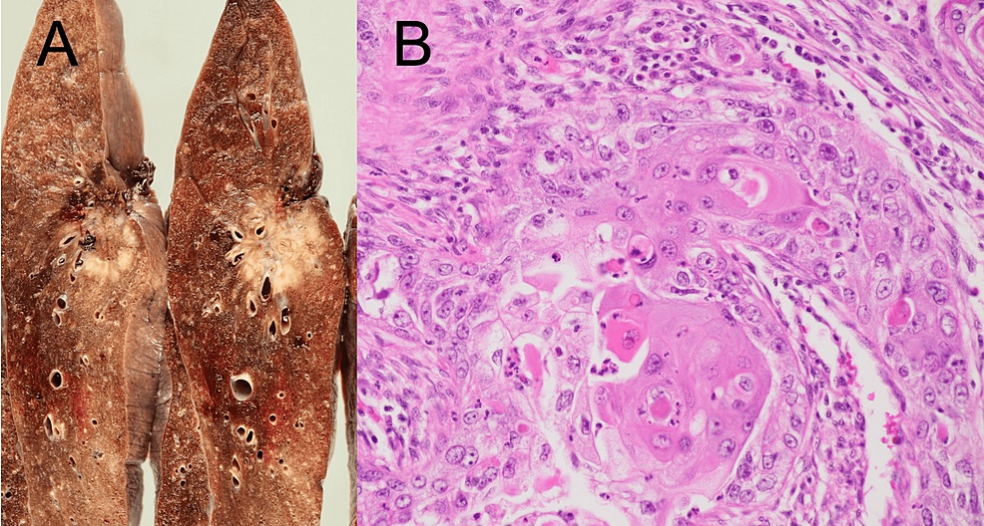
Right lung resection specimen and pathological findings. The resected specimen showed a relatively well-circumscribed white lesion (A). Pathological findings revealed invasive proliferation of atypical cells, cancer pearls, and cancer nests characteristic of squamous cell carcinoma (B).

**Figure 5:**
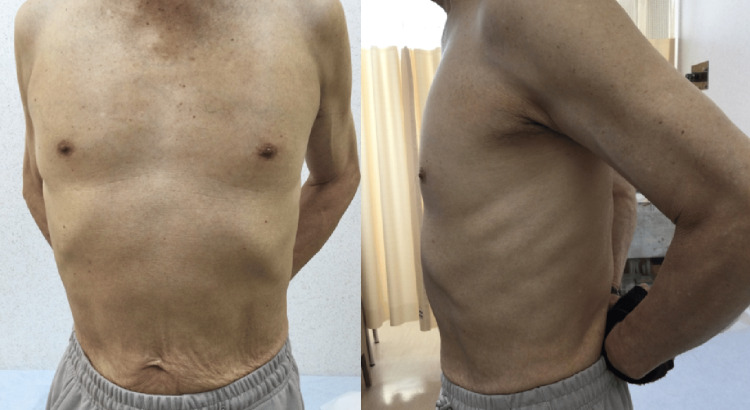
Disappearance of the left upper abdominal bulging. Approximately one week after lung cancer surgery, the left upper abdominal bulging spontaneously resolved.

A 71-year-old male presented with left upper abdominal bulging and pain. He had noticed the symptoms three days prior and visited a local clinic. A CT scan revealed muscle relaxation at the site of the bulge and incidentally detected a nodule in the right lower lobe suspicious for lung cancer, prompting referral to our hospital. His medical history included type 2 diabetes mellitus and dyslipidemia. Physical examination on admission revealed extensive bulging and pain in the left upper abdomen (Figure 1), but no other abnormal findings, including skin lesions. Abdominal CT showed muscle relaxation at the site of the left upper abdominal bulge but no obvious hernia (Figure 2). Chest CT revealed an irregular nodule in the right lower lobe (Figure 3) without findings suggestive of mediastinal or hilar lymph node metastasis, leading to a suspected diagnosis of right lower lobe lung cancer cT2aN0M0 Stage IB. The patient underwent right lower lobectomy by single-port video-assisted thoracoscopic surgery (VATS), and the final pathological diagnosis was squamous cell carcinoma pT2aN0M0 Stage IB (Figure 4). One week after surgery, the left upper abdominal bulging spontaneously resolved, leading to a diagnosis of abdominal pseudohernia (Figure 5).

## Discussion

We experienced a case of a pseudoabdominal wall hernia that disappeared after lung cancer surgery. Abdominal wall hernias occur when intra-abdominal organs protrude through weakened areas of the abdominal wall due to surgery or other causes, and those without a hernia sac are called pseudoabdominal wall hernias [1]. Known causes of pseudoabdominal wall hernias include spinal disorders such as intervertebral disc herniation, surgery, trauma, and diabetes [2], but cases associated with herpes zoster are frequently reported.

This patient was undergoing oral treatment for type 2 diabetes. While there are reports of poorly controlled type 2 diabetes patients developing pseudohernias due to diabetic neuropathy [3], there was no worsening of diabetes control before or after onset, and no other findings suggestive of diabetic neuropathy were observed. In this case, there was no history of herpes virus manifestation. In the absence of other clear causes that could explain it alone, we considered the involvement of herpes zoster. Herpes zoster typically presents with rash and systemic symptoms in the affected skin segment, but motor complications occur in about 5% of patients, and pseudoabdominal wall hernias are reported to occur in less than 1% [4].

The mechanism by which herpes zoster causes pseudoabdominal wall hernias is understood to be muscle relaxation of the affected muscles due to the herpes zoster virus activating in the ganglia and causing nerve damage [4]. In this case, we thought that nerve damage caused by herpes zoster led to a relaxation of the left oblique abdominal muscles, resulting in a pseudoabdominal wall hernia in the left lateral abdomen. There are reports of herpes zoster reactivation cases without rash or pain [5] and cases of pseudohernias without preceding rash [6]. However, the lack of direct evidence for herpes zoster involvement can be considered a limitation of this case.

No immunological parameter measurements were conducted this time. The diagnosis was made based on the location commonly affected by pseudohernia and the bulging morphology matching CT findings. PD-L1 status was also not assessed due to insurance limitations.

**Figure 6:**
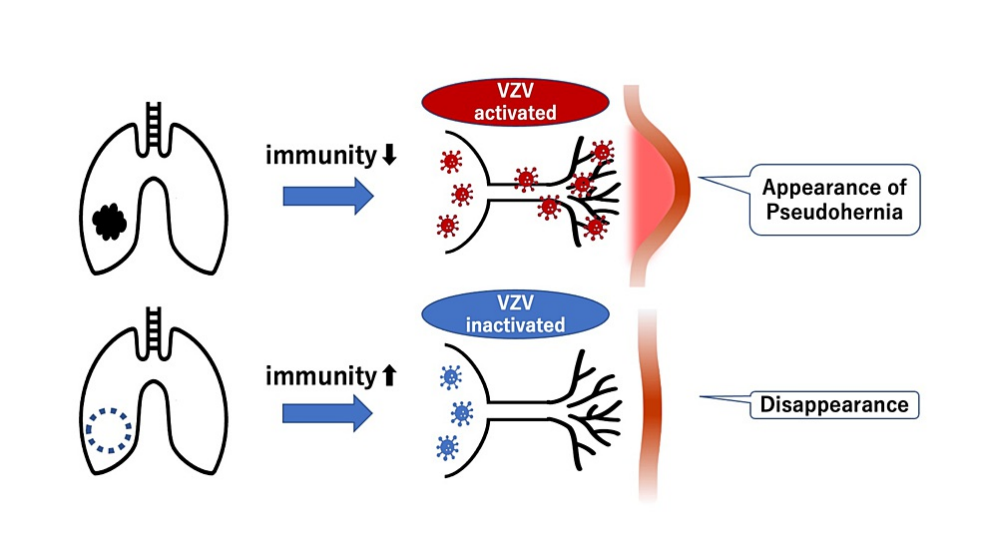
Immunity and pseudohernia mechanism. Top panel: Decreased immunity activates varicella-zoster virus (VZV), leading to pseudohernia appearance. Bottom panel: Increased immunity inactivates VZV, causing pseudohernia disappearance. This figure is an original creation by the author(s) and does not require external permission for reproduction.

A method to confirm herpes zoster reactivation is to quantify varicella-zoster virus (VZV) DNA in blood using polymerase chain reaction (PCR). PCR has been shown to be a powerful tool complementing clinical diagnosis based on symptoms, and blood VZV DNA levels are reported to be useful for assessing disease severity and treatment efficacy [7]. As the hernia disappeared one week after lung cancer surgery, we thought that immune dysregulation due to lung cancer caused herpes zoster virus reactivation, resulting in the pseudoabdominal wall hernia. It is known that in a cancer-bearing state, tumors induce immunosuppressive cells such as regulatory T cells (Tregs) and myeloid-derived suppressor cells (MDSCs), and secrete immunosuppressive cytokines such as transforming growth factor-beta (TGF-β) and interleukin 10 (IL-10) to evade attack from the immune system, causing decreased cellular immunity in patients [8]. It is also known that decreased cellular immunity contributes to herpes zoster virus reactivation [9]. Therefore, we thought that the immune dysregulation improved to some extent after lung cancer surgery, the activity of the herpes zoster virus decreased, and the hernia spontaneously resolved (Figure 6).

As previous reports have stated, pseudoabdominal wall hernias do not require surgical intervention [1,10], and it is important to prioritize the treatment of herpes zoster or the underlying disease. In this case, lung cancer was incidentally found on chest CT at a previous hospital, but normally, chest CT is rarely performed for patients presenting with only abdominal swelling, nor can it be said that there is a need for it. On the other hand, when encountering pseudoabdominal wall hernias, we believe it is important to search for causes with the possibility of underlying malignant tumors, collagen diseases, or HIV in mind. Also, while this case lacked direct evidence of herpes zoster involvement, if there is no other clear cause, quantifying VZV DNA by PCR may be helpful for diagnosis.

## Conclusions

Abdominal pseudohernia resulting from herpes zoster reactivation was noted following lung cancer surgery and resolved spontaneously post surgery, suggesting an improvement in immune function. The occurrence of herpes zoster, which may present with or without a rash, necessitates vigilant observation. It is crucial for dermatologists and surgeons to be aware of this condition to ensure effective diagnosis and management.
